# *In silico* analysis of antidiabetic potential of phenolic compounds from blue corn (*Zea mays* L.) and black bean (*Phaseolus vulgaris* L.)

**DOI:** 10.1016/j.heliyon.2020.e03632

**Published:** 2020-03-27

**Authors:** K. Damián-Medina, Y. Salinas-Moreno, D. Milenkovic, L. Figueroa-Yáñez, E. Marino-Marmolejo, I. Higuera-Ciapara, A. Vallejo-Cardona, E. Lugo-Cervantes

**Affiliations:** a Center for Research and Assistance in Technology and Design of the State of Jalisco, A.C. (CIATEJ), Food Technology Unit, 45019 Jalisco, Mexico; b Instituto Nacional de Investigaciones Forestales, Agrícolas y Pecuarias (INIFAP), Tepatitlán 47600, Jalisco, Mexico; c Department of Internal Medicine, UC Davis School of Medicine, University of California, Davis, USA; d Université Clermont Auvergne, INRAE, UNH, F-63000 Clermont–Ferrand, France

**Keywords:** Food science, Nutrition, Natural product chemistry, Diabetes, Polyphenols, Black bean, Blue corn, Molecular docking

## Abstract

The growing interest in bioactive compounds, especially in polyphenols, is due to their abundance in the human diet and potentially positive effects on health. The consumption of polyphenols has been shown to possess anti-diabetic properties by preventing insulin resistance or insulin secretion through different signaling pathways, this effect is associated with their capacity to exert genomic modulations. Several studies have suggested that polyphenols could also bind to cellular proteins and modulate their activity, however, the mechanisms of action underlying their beneficial effects are complex and are not fully understood. The aim of this work was to characterize phenolic compounds present in blue corn and black bean extracts as well as identify their potential interactions with target proteins involved in diabetes pathogenesis using *in silico* approach. Total polyphenols content of both blue corn and black beans was identified using UPLC-ESI/qTOF/MS and quantified by colorimetric assays. In this work we identified twenty-eight phenolic compounds in the extracts, mainly anthocyanins, flavonols, hydroxycinamic acids, dihydroxybenzoic acids, flavones, isoflavones, and flavanols. Interactome of these compounds with thirteen target proteins involved in type 2 diabetes mellitus was performed *in-silico*. In total, 312 bioactive compounds/protein interaction analyses were acquired. Molecular docking results highlighted that nine of the top ten interactions correspond to anthocyanins, cyanidin 3-glucoside with 11β-HS, GFAT, PPARG; delphinidin 3-glucoside with 11β-HS, GFAT, PTP and RTKs; and petunidin 3-glucoside with 11β-HS and PTP. These proteins are involved in mechanisms regulating functions such as inflammation, insulin resistance, oxidative stress, glucose and lipid metabolism. In conclusion, this work provides a prediction of the potential molecular mechanism of black bean and blue corn polyphenols, specifically anthocyanins and could constitute new pathways by which compounds exert their antidiabetic benefits.

## 1 Introduction

Poor dietary habits and sedentary lifestyle have increased the prevalence of chronic degenerative diseases, such as type 2 diabetes mellitus (T2DM) (Mellendick et al., 2018). The International Federation of Diabetes estimated that 425 million people were diagnosed with T2DM around the world. One of the main problems in diabetes is the growing complications after diagnostic, including retinopathy, neuropathy, nephropathy, cardiovascular diseases, skin complications. This represents an important economic burden since 12% of global health expenditure is spent in diabetic population (International Diabetes Federation, 2017).

T2DM is a complex disease characterized by high glucose plasmatic levels. It involves different cellular pathways such as insulin secretion, insulin resistance, carbohydrate absorption. Some of the proteins identified as playing an important role in development of T2DM is glucokinase, AMP-activated protein kinase, 11 β-hydroxysteroid dehydrogenase, insulin receptor substrate, interleukin 1 beta, dipeptidyl peptidase IV, C-reactive protein, glutamine fructose-6-phosphate amidotransferase, peroxisome proliferator activated receptor gamma, protein tyrosine phosphatases, tyrosine kinase insulin receptor, protein kinase B and insulin receptor. Different medical strategies have been developed to help fight T2DM such as dietary modifications and exercise plus antidiabetic and anti-obesity medications. Nevertheless, medical treatment recurrently has adverse effects on patients (DeFronzo, 2009). The knowledge of new therapeutic targets as well as new antidiabetic compounds with less adverse effects and greater efficacy is necessary to prevent and control T2DM.

A healthy lifestyle adherence that includes a high-quality diet, regular exercise, and maintenance of adequate body weight, are strongly associated with a best management of T2DM (Wang and Hu, 2018). One of the most important lifestyle factors is the diet. Epidemiological studies have shown that diets with consumption patterns rich in fruits and vegetables could have an impact on glucose levels in diabetic patients (Mellendick et al., 2018; Ramírez-Silva et al., 2009; Villani et al., 2018). Polyphenols are one of the most abundant group of bioactive compounds in plants, fruits and vegetables. They are secondary plant metabolites and can confer diverse organoleptic properties such as bitterness, astringency, color or flavor. Different epidemiological, animal and clinical studies have revealed that they exert health effect properties, such as prevention of cardiovascular diseases or neurodegenerative disorders. Studies have been conducted to investigate the effect of polyphenols on T2DM (Aryaeian et al., 2017), such as catechins and isoflavones. It has been observed that these compounds can improve muscle vascular and peripheral insulin resistance, respectively, and an increase in insulin sensitivity in T2DM patients (Cao et al., 2018). Other compounds such as stilbenoids, specifically resveratrol, have shown to affect β pancreatic cell functions, reduce blood glucose levels, or exert anti-inflammatory and anti-oxidative effects (Kim et al., 2014). Anthocyanins, a subclass of flavonoids, have been shown to have diverse mechanisms of action, such as glucose homeostasis through β cell mass and function, insulin sensitivity and glucose uptake, besides lower levels of inflammatory markers in serum (Guo et al., 2016; Yang and Zhai, 2010). Proanthocyanidins have been shown to inhibit α-glucosidase activity similar to drugs mechanism (Mojica et al., 2017) and regulate glucose levels across GLUT 4 translocation. At last, phenolic acids were associated with the improvement of fasting glucose levels and glucose uptake in skeletal muscle (Xiao and Hogger, 2015).

Common beans and corn are one of the most consumed legumes in the world. Regarding black beans, Mexico produces 22% of the total world production and black bean variety is usually consumed in the Mexican central and south regions (Salinas-Moreno et al., 2005). Nutritionally, beans are a good source of proteins, complex carbohydrates, dietary fibers, iron, and other minerals as well as vitamins (Lin et al., 2008). On the other hand, corn represents 38.6% of Mexican cultivable territory and is the main product consumed by Mexican population. The nutrient composition of corn is mainly of carbohydrates, proteins, dietary fiber and fat (Vázquez-Carrillo et al., 2018). Nowadays, 59 varieties of blue corn have been described (Salinas-Moreno et al., 2005).

The interest in blue corn and black bean has been increased by the significant number of polyphenols, since it has been reported that blue corn contains between 918.9 to 1165.5 mg/kg DW (GAE) (Huang et al., 2015; Ramos-Escudero et al., 2012; Salinas-Moreno et al., 2005; Urias-Lugo et al., 2015; Yang and Zhai, 2010) and black bean between 5.4 to 18.3 mg EAG/g mg/kg DW (GAE) (Cardador-Martínez et al., 2002; de Mejıa et al., 1999; Mojica et al., 2015). It has been reported that phenolic compounds have a positive effect on T2DM (Anhê et al., 2013; Cao et al., 2018; Jung et al., 2014; Kim et al., 2016a, Kim et al., 2016b; Rasouli et al., 2017; Rocha et al., 2019). The mechanism action of these compounds can occur throughout different manners: first across their metabolism in a variety of pathways acting on changing the concentration of substrates or intermediates modifying cell signaling; second, working as a ligand for transcription factors; and third interacting directly with signaling pathways. All of the mentioned mechanisms will produce modifications in gene expression that will affect positively the principal organs and tissues (pancreas, adipose tissue, liver and muscle) involved in T2DM (Berná et al., 2014).

It has been reported that both foods have different beneficial activities on health, such as antioxidant, anti-inflammatory or, anti-mutagenic, among others. Moreover, they enhance insulin production and glucose homeostasis, but in spite of this knowledge, less is described about the mechanisms and target proteins involved in regulation of glucose levels, insulin resistance, insulin production and inflammation (Del Pozo-Insfran et al., 2006). Mechanisms of action identified as underlying the observed anti-diabetic effects are insulin secretion, insulin resistance, and carbohydrate absorption. The search of new therapeutic targets remains a challenge, although there are many different groups of polyphenols for diabetes treatment that have not been investigated thoroughly. Taken together, the aim of this work was to investigate the total polyphenol content of black bean and blue corn extracts and asserts the potential antidiabetic properties using *in silico* approach by predicting the binding interactions between polyphenols with target cell signaling proteins involved in the development of diabetes.

## 2 Materials and methods

### 2.1 Extracts preparation

The sample of black bean was obtained from Durango, México and blue corn from Jalisco, México. The extraction procedure was as follows: 100 g of each sample was finely ground to obtain flours. Both flours were mixed separately, in a solution of ethanol (99.9%) with clorhidric acid (.1%) in a pot with stir for 4 h at room temperature and covered from light. The mixtures were centrifuged for 20 min at 13000 rpm, the supernatant was decanted and rota-evaporated at 38 °C at 90 rpm until ethanol was completely removed. Following this step, the extracts were frozen at -20 °C overnight and lyophilized for three days at -50 °C and 250 mBar. The dried extracts were conserved at 4 °C until their use.

### 2.2 Determination of total phenolic concentration

To measure the total phenolic concentration, the Folin-Ciocalteu method was employed (Rover and Brown, 2013). The acidified ethanolic extract was mixed with the Folin-Ciocalteu reagent and allowed to rest for 6 min after which Na2CO3 was added. The volume was adjusted to 3 ml with distilled water, samples were shaken in a vortex and stored for 90 min at room temperature (22°C ± 2 °C) in the dark. The samples were centrifuged, and the absorbance was measured in a spectrophotometer (Perkin-Elmer Lambda 25 UV/Vis, Waltham MA, USA) at 760 nm. Total soluble phenols were calculated based on a gallic acid curve and expressed as gallic acid equivalents (GAE) kg-^1^ of dry sample (Salinas-Moreno et al., 2012).

### 2.3 Total flavonoids concentration

The methodology was determined according to Woisky and Salatino (1998), modified by Sumczynski et al. (2015) using 8.5 mL of 20% ethanol that was mixed with 0.85 mL of the extract and 0.375 mL of 0.5 M NaNO_2_. After 5 min, 0.375 mL of 0.3 M AlCl3·6 H2O solution was added, and the mixture was allowed to stand for 5 min before adding 2.5 mL of 1 M NaOH. The absorbance was measured after 10 min at 506 nm (Perkin-Elmer Lambda 25 UV/Vis, Waltham MA, USA). Rutin was used as a standard and the results were expressed as mg of rutin equivalent (RE) per kg of the sample (mg/kg RE sample).

### 2.4 Total anthocyanins concentration

Total anthocyanins were quantified according to the method by Salinas-Moreno et al. (2012). A standard curve of cyanidin 3-glucoside (Extrashintase, France) was prepared and the absorbance of the extracts was measured at 520 nm in a spectrophotometer (Perkin-Elmer Lambda 25 UV/Vis, Waltham MA, USA). The total content of the samples was expressed in mg equivalent of cyanidin 3-glucoside (ECG) kg^−1^ of the dry sample.

### 2.5 Quantification of total proanthocyanidins

The 4-dimethylaminocinnamaldehyde (DMAC) assay was performed to quantify total proanthocyanidins. Briefly, a mixture of 2% DMAC in methanol (w/v) in 6N H_2_SO_4_ (50:50 v/v) was prepared. Then, 20 μl of the sample was added to 2380 μl of methanol and mixed with 100 μl of DMAC in 3-mL disposable plastic cuvettes (path length = 1 cm). The mixture was allowed to stand for 25 min in dark and the absorbance was measured at 640 nm (Perkin-Elmer Lambda 25 UV/Vis, Waltham, MA. USA). A curve of catechin was prepared and the results were expressed as mg equivalent of catechin (mg/kg EC) (Wallace and Giusti, 2010).

### 2.6 Phenolic compounds identification by UPLC-ESI/qTOF/MS

The extracts were characterized by ultra-performance liquid chromatography-electrospray ionization/quadrupole-time-of-flight high definition mass spectrometry technique (UPLC-ESI/qTOF/MS). Both samples were filtered (0.22 μm) with an injection volume of 3 μL, by using a BEH C18 1.7 μm column (Acquity UPLC, Waters, Mildford MA, USA) at 30 °C, the flow rate was of 0.3 mL/min with the next solvent system: acetonitrile (solvent A) and Water pH 2.5 with trifluoracetic acid (solvent B) 15 % at 0 min, 35 % A at 6.97 min, 35 % at 15 min. Then, the samples were nebulized by ESI-Q-ToF (Xevo G2-XS QTof, Waters, Milford MA, USA) and the ions were collected between 100 to 1500 Da, in positive (+) mode with the MassLynx software (v4.1, Waters, Milford, MA, USA). All the data were analyzed in the MestReNova software (v12.0.20910, Mestrelab Research S.L.) (Anguiano-Sevilla et al., 2018). The ESI conditions were a positive ion mode capillary voltage 32 Kv; SAMPLIng cone 42; source offset 80; and N2 cone gas flows: cone gas 50 1/h: desolvation gas 5000 MS/MS cone voltage: 35V and energy collision: 25V dry gas (Carballo-Uicab et al., 2019).

### 2.7 Molecular docking analysis

Molecular docking studies were employed to explore the potential interaction/binding between ligands and proteins involved in T2DM. Twenty-four ligands were selected according to the results of UPLC-ESI/qTOF/MS that represent the major compounds found in the extracts. Metformin and sitagliptin were used as controls. On the other hand, we investigated the principal target proteins that participate in diabetes pathogenesis. In total, 13 proteins were identified. Their three-dimensional structure was obtained from the Protein Data Bank (PDB) database and their function was acquired from the literature (Table 1). Docking calculations were carried out using DockingServer (https://www.dockingserver.com/) (Bikadi and Hazai, 2009) and BIOVIA Discovery Studio 2017 (http://www.3dsbiovia.com/) for designing.

**Table 1:** Function of target proteins involved in type 2 diabetes mellitus.

Protein name	Abbreviation	PDB code	Function
Glucokinase	GK	1V4S	Catalyzes the transfer of phosphate from ATP to glucose to generate glucose 6-phosphate (Nguyen and Le, 2012).
AMP-activated protein kinase	AMPK	2H6D	Involved in the stimulation of glucose transport and fatty acid oxidation (Nguyen and Le, 2012)
11 β-hydroxysteroid dehydrogenase 1	11β-HS1	1BHS	Produces insulin resistance through the conversion of cortisone to cortisol (Nguyen and Le, 2012; Rathore et al., 2016).
Insulin receptor substrate	IRS	1K3A	Impairment of IRS-2 signaling in the β-cell produces β-cell loss in T2DM (Rathore et al., 2016).
Interleukin 1 beta	IL-1	9ILB	Contributes to inflammation of beta cells in pancreas (DeFronzo, 2009).
Dipeptidyl peptidase IV	DPPIV	1J2E	Inhibits the action of GIP and GLP-1, increasing glucose levels (Nguyen and Le, 2012).
C-reactive protein	CRP	1GNH	Involved in chronic inflammation in adipose tissue and leads to insulin resistance (DeFronzo, 2009).
Glutamine fructose-6-phosphate amidotransferase	GFAT	2ZJ3	Increased the flux of glucose through the pathway where GFAT is a key catalyst that can lead to insulin resistance (Nguyen and Le, 2012; Rathore et al., 2016).
Peroxisome proliferator activated receptor gamma	PPARG	1PRG	Plays a key role in adipogenesis (Nguyen and Le, 2012).
Protein tyrosine phosphatases	PTP	2NT7	Removes phosphate groups from phosphorylated tyrosine residues on proteins in liver and fat (Rathore et al., 2016).
Tyrosine kinase insulin receptor	RTKs	1IRK	Plays a key role on insulin signaling pathway (Nguyen and Le, 2012).
Protein kinase B	PKB	3E87	May contribute to β-cell loss in the pathogenesis of type 2 diabetes (Rathore et al., 2016).
Insulin receptor	IR	2HR7	Controls access to blood glucose in body cells (Nguyen and Le, 2012).

#### 2.7.1 Protein preparation for dockings

Docking calculations were carried out on 13 proteins models involved in T2DM pathogenesis. The crystalline structures were downloaded from Protein Data Bank website (https://www.rcsb.org/pdb/home/home.do) and saved in pdb format. The ID's of selected proteins are listed below (Table 1). Essential hydrogen atoms, Kollman united atom type charges, and solvation parameters were added with the aid of AutoDock tool. Affinity (grid) maps of 20 × 20 × 20 Å grid points and 0.375 Å spacing were generated using the Autogrid program. AutoDock parameter set- and distance-dependent dielectric functions were used in the calculation of the van der Waals and the electrostatic terms, respectively (Morris et al., 1998).

#### 2.7.2 Ligand and energy minimization

The MMFF94 force field (Halgren, 1998) was used for energy minimization of ligands molecules using DockingServer. Gasteiger partial charges were added to the ligand atoms. Non-polar hydrogen atoms were merged, and rotatable bonds were defined. The ligands were downloaded from the PubChem software (https://pubchem.ncbi.nlm.nih.gov).

#### 2.7.3 Ligand-protein docking

Molecular docking studies were carried out using the parameters of molecular docking software (https://www.dockingserver.com). Docking simulations were performed using the Lamarckian genetic algorithm (LGA) and the Solis & Wets local search method (Solis and Wets, 1981). Initial position, orientation, and torsions of the ligand molecules were set randomly. Each docking experiment was derived from 100 different runs that were set to terminate after a maximum of 2,500,000 energy evaluations. The population size was set to 150. During the search, a translational step of 0.2 Å, quaternion and torsion steps of 5 were applied.

#### 2.7.4 Statistical analysis

Standard means and deviations were performed using the SPSS software version 19.0 (IBM **SPSS** Statistics for Windows, version 19.0. IBM Corp., Armonk, N.Y., USA).

## 3 Results

### 3.1 Content of total polyphenols, flavonoids, anthocyanins and proanthocyanidins

**Figure 1:**
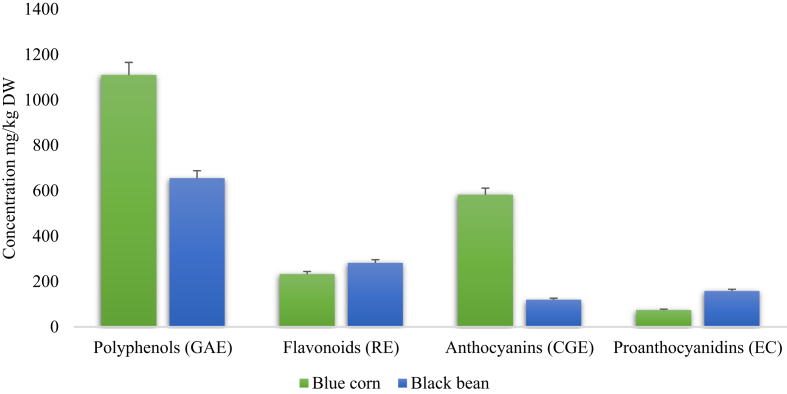
Total soluble polyphenols, flavonoids, anthocyanins and proanthocyanidins content in blue corn and black bean extracts. GAE: gallic acid equivalents; RE: rutin equivalents; CGE: cyanidin 3-glucoside equivalents; CE: catechin equivalents.

The polyphenol content of both corn and bean is shown in Figure 1. Blue corn extract (BCE) demonstrate a polyphenols content of 1110.05 ± 4.07 mg/kg DW (GAE) and the black bean extract (BBE) showed a lower amount with 655.55 ± 1.31 mg/kg DW (GAE). Regarding flavonoids content, BBE had a lower content 233.0 ± 8.50 mg/kg DW (RE) than BCE 282.31 ± 0.01 mg/kg DW (RE). Moreover, the anthocyanins are in higher proportion in BCE than BBE with 582.47 ± 2.03 mg/kg DW (CGE) and 120.31 ± 1.31 mg/kg DW (ECG) respectively. At last, proanthocyanidins proportion turned out to be higher in BBE 158.24 ± 0.01 with respect to BCE with 74.65 ± 0.01 mg/kg DW (CE).

### 3.2 Phenolic compounds identified by UPLC-ESI/qTOF/MS

**Figure 2:**
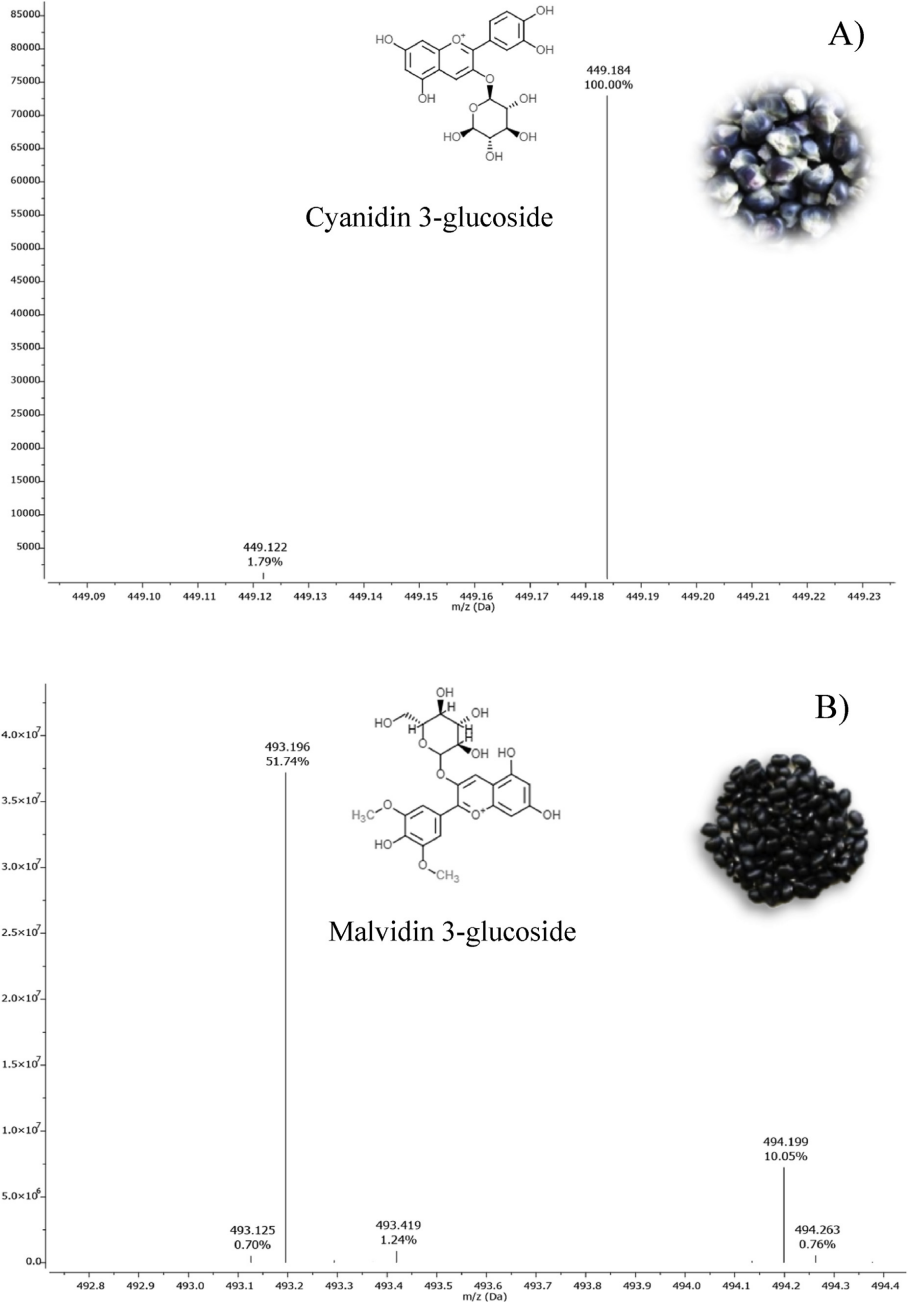
UPLC ESI/qTOF/MS identification. A) MS spectrum of blue corn extract with cyanidin 3-glucoside detection (*m/z* 449.184), B) MS spectrum of black bean extract with malvidin 3-glucoside detection (*m/z* 493.196).

**Figure 3:**
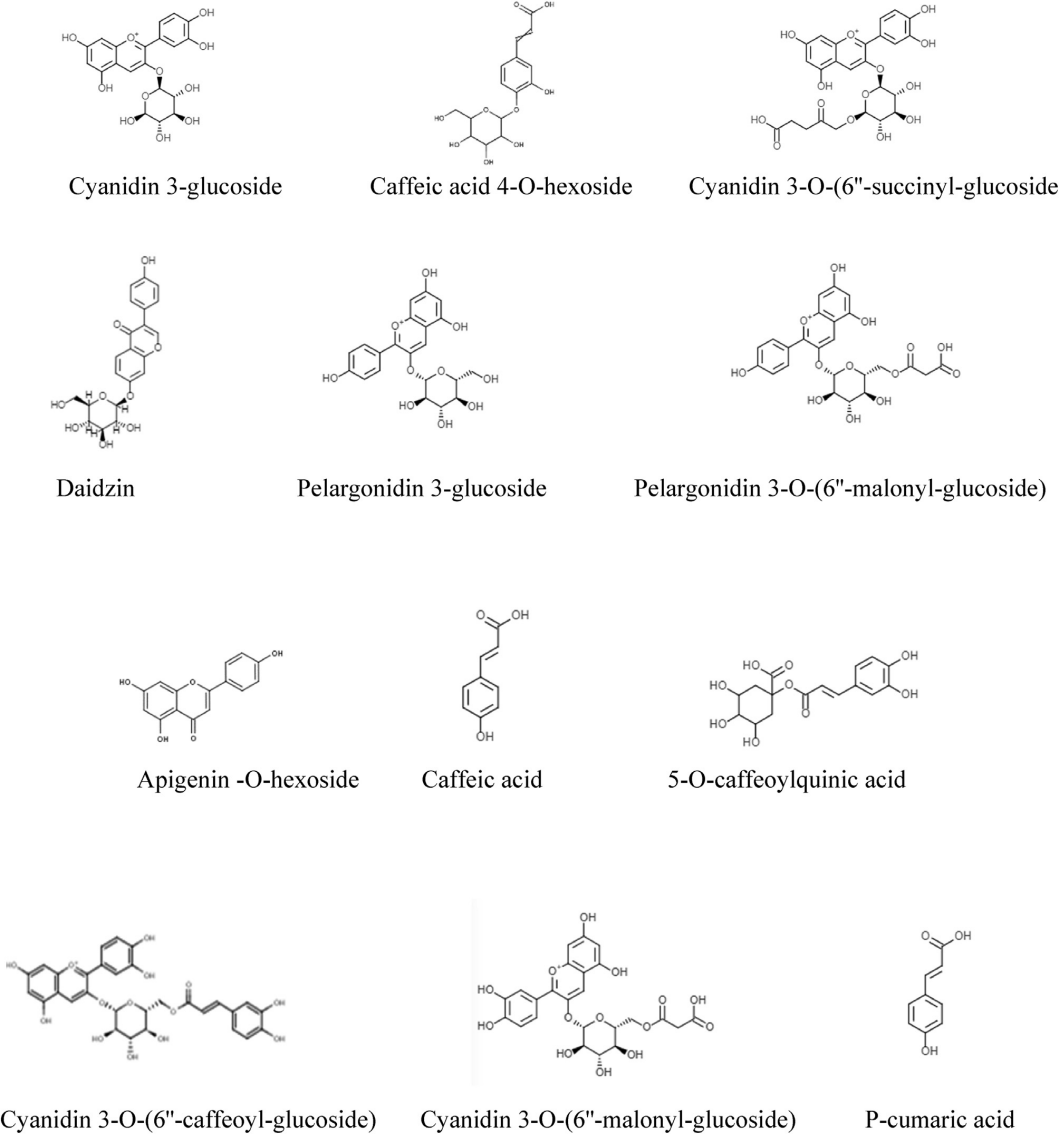
Chemical structures of polyphenols identified in blue corn extract used for *in silico* analyses.

**Figure 4:**
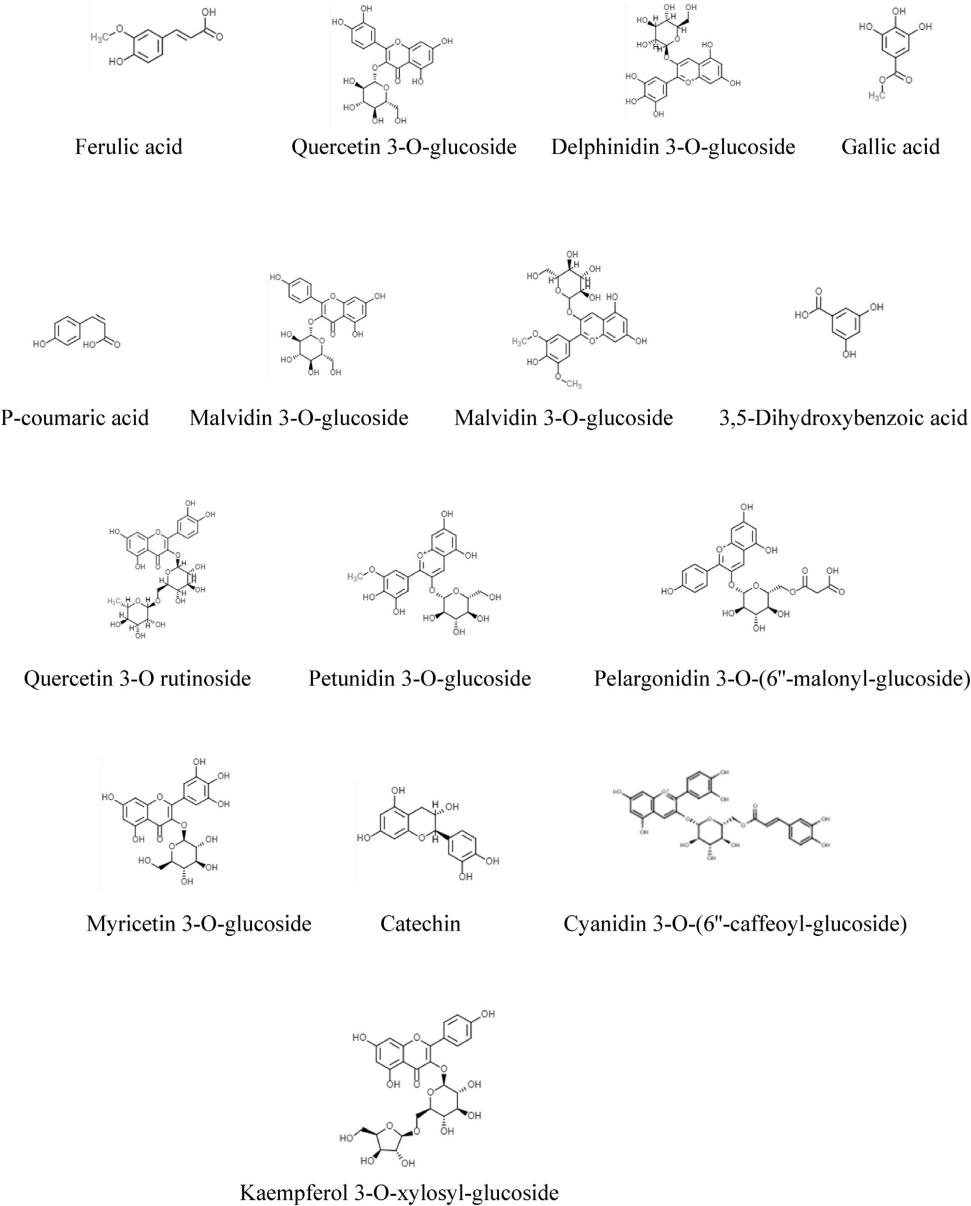
Chemical structures of polyphenols identified in black bean extract used for *in silico* analyses.

Regarding the identification of polyphenols by UPLC-ESI/qTOF/MS, compounds were characterized according to their m/z value, and retention time as compared to previous reports of other studies (González-Manzano et al., 2008; Abdel-Aal et al., 2006; Hernández et al., 2018; Lee et al., 2018; Koh et al., 2014; Hart et al., 2015; Pitura and Arntfield, 2019; Mojica et al., 2015) as shown in Table 2 and Figure 2. Altogether, Figure 3 is showing the structures of the twelve polyphenols identified in BCE. Six of them belong to anthocyanins: cyanidin 3-glucoside, cyanidin 3-O-(6″-succinyl-glucoside), pelargonidin 3-glucoside, pelargonidin 3-O-(6″-malonyl-glucoside), cyanidin 3-O-(6″-caffeoyl-glucoside) and cyanidin 3-O-(6″-malonyl-glucoside); four to hydroxycinnamic acids: caffeic acid 4-O-hexoside, caffeic acid, 5-O-caffeoylquinic acid and p-cumaric acid; one isoflavone: daidzin; and one flavone: apigenin -O-hexoside. Likewise, structures of fifteen compounds identified in BBE are shown in Figure 4, six correspond to anthocyanins: Delphinidin 3-O-glucoside, malvidin 3-O-glucoside, petunidin 3-O-glucoside, pelargonidin 3-O-(6″-malonyl-glucoside), myricetin 3-O-glucoside and cyanidin 3-O-(6″-caffeoyl-glucoside); three to hydroxycinnamic acids: ferulic acid, gallic acid, and p-coumaric acid; four to flavonols: quercetin 3-O-glucoside, kaempferol 3-O-glucoside, quercetin 3-O rutinoside and kaempferol 3-O-xylosyl-glucoside; one flavanol: catechin; and one dihydroxybenzoic acid: 3,5- Dihydroxybenzoic acid.

**Table 2:** Main polyphenols identified in blue corn and black bean extracts by UPLC ESI/qTOF/MS and used as ligands in molecular docking analysis.

Proposed molecule	Abbreviation	Rt (min)	*m/z*	Condensed formula	Polyphenol group	References
Cyanidin 3-glucoside	C3G	1.01	449.184	C21H21O11	Anthocyanin	(Abdel-Aal et al., 2006; González-Manzano et al., 2008)
Caffeic acid 4-O-hexoside	C4H	1.52	342.298	C15H18O9	Hydroxycinnamic acid	(Hernández et al., 2018)
Cyanidin 3-O-(6″-succinyl-glucoside)	C3S	1.67	549.214	C25H25O14	Anthocyanin	(Abdel-Aal et al., 2006)
Daidzin	DAD	1.84	438.314	C21H20O9	Isoflavone	(Hernández et al., 2018)
Pelargonidin 3-glucoside	P3G	1.86	475.406	C21H21O10	Anthocyanin	(Abdel-Aal et al., 2006; González-Manzano et al., 2008)
Pelargonidin 3-O-(6″-malonyl-glucoside)	P3M	4.25	520.427	C24H23O13	Anthocyanin	(González-Manzano et al., 2008)
Apigenin -O-hexoside	AOH	4.62	523.446	C21H18O11	Flavone	(Hernández et al., 2018)
Caffeic acid	CFA	5.48	263.289	C9H8O3	Hydroxycinnamic acid	(Hernández et al., 2018)
5-O-caffeoylquinic acid	CQA	5.57	355.348	C16H18O9	Hydroxycinnamic acid	(Hernández et al., 2018)
Cyanidin 3-O-(6″-caffeoyl-glucoside)	CCG	5.65	612.282	C30H27O14	Anthocyanin	(González-Manzano et al., 2008)
Cyanidin 3-O-(6″-malonyl-glucoside)	CMG	5.95	536.254	C24H23O14	Anthocyanin	(Abdel-Aal et al., 2006; González-Manzano et al., 2008)
P-cumaric acid	PCA	6.00	243.097	C9H8O3	Hydroxycinnamic acid	(Hernández et al., 2018)
Ferulic acid	FRA	0.41	217.113	C10H10O4	Hydroxycinnamic acid	(Lee et al., 2018)
Quercetin 3-O-glucoside	Q3G	0.81	465.162	C21H20O12	Flavonol	(Lee et al., 2018; Koh et al., 2014)
Delphinidin 3-O-glucoside	D3G	0.91	465.165	C21H21O12	Anthocyanin	(Lee et al., 2018; Hart et al., 2015)
Gallic acid	GAA	1.01	188.111	C7H6O5	Hydroxycinnamic acid	(Lee et al., 2018; Hart et al., 2015)
P-coumaric acid	PCA	1.34	164.111	C9H8O3	Hydroxycinnamic acid	(Hart et al., 2015; Pitura and Arntfield, 2019)
Kaempferol 3-O-glucoside	K3G	1.42	493.196	C21H20O11	Flavonol	(Mojica et al., 2015)
Malvidin 3-O-glucoside	M3G	1.51	493.196	C23H25O12	Anthocyanin	(Pitura and Arntfield, 2019)
3,5-Dihydroxybenzoic acid	DHA	2.18	177.094	C7H6O4	Dihydroxybenzoic acid	(Koh et al., 2014)
Quercetin 3-O rutinoside	Q3R	3.81	811.605	C33H40O21	Flavonol	(Mojica et al., 2015)
Petunidin 3-O-glucoside	PTG	4.09	518.411	C22H23O12	Anthocyanin	(Hart et al., 2015; Pitura and Arntfield, 2019)
Pelargonidin 3-O-(6″-malonyl-glucoside)	P3M	4.40	520.427	C24H23O13	Anthocyanin	(Mojica et al., 2015)
Myricetin 3-O-glucoside	MYG	4.70	522.441	C21H20O13	Anthocyanin	(Mojica et al., 2015; Pitura and Arntfield, 2019)
Catechin	CAT	5.23	291.287	C15H14O6	Flavanol	(Koh et al., 2014; Mojica et al., 2015; Pitura and Arntfield, 2019)
Cyanidin 3-O-(6″-caffeoyl-glucoside)	CCG	5.65	612.281	C30H27O14	Anthocyanin	(Lee et al., 2018; Hart et al., 2015)
Kaempferol 3-O-xylosyl-glucoside	K3X	6.26	663.557	C26H28O15	Flavonol	(Lee et al., 2018)

### 3.3 Molecular docking results

**Figure 5:**
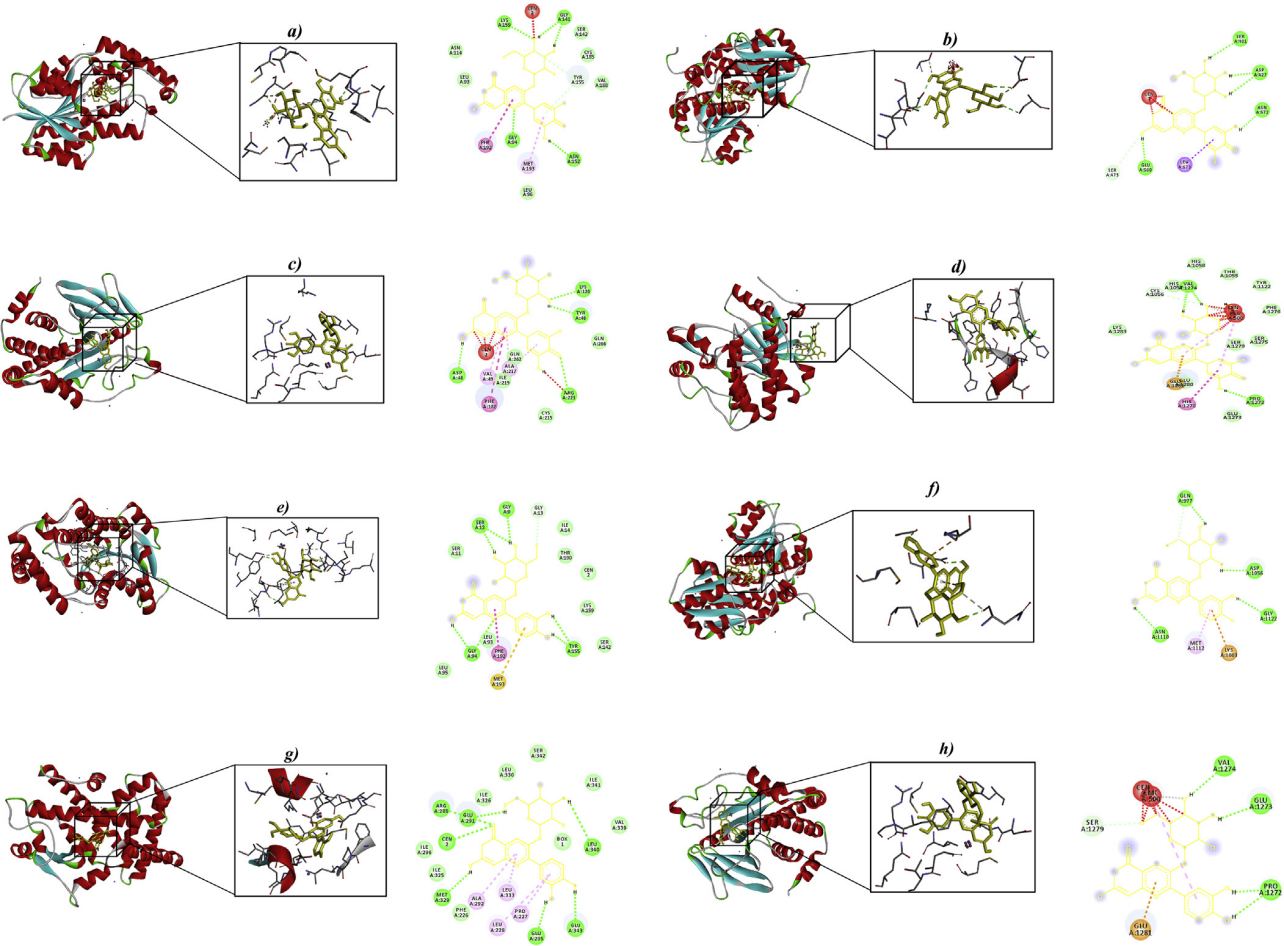
Binding of ligands from blue corn and black bean with the best interactions. The boxes indicate the binding region. a) D3G + 11β-HS, b) D3G + GFAT, c) D3G + PTP, d) D3G + RTK's, e) C3G + 11β-HS, f) C3G + GFAT, g) C3G + PPARG, h) P3G + PTP.

The compounds of each extract were used to run the dockings. Molecular docking analysis was carried out with thirteen proteins (1V4S, 2H6D, 1BHS, 1K3A, 9ILB, 1J2E, 1GNH, 2ZJ3, 1PRG, 2NT7, 1IRK, 3E87, and 2HR7) identified from the literature as playing important role in the pathogenesis of T2D. In total, 312 *in-silico* docking analyses were performed. Their free energy binding is showed in Table 3. The ten best molecular interactions were chosen based on their inhibitory potential, free energy expressed in kcal/Mol, inhibition constant (Ki) expressed in μM, number of hydrogen bonds and interacting residues (Table 4). Our results suggest that in total, nine of ten best interactions belong to anthocyanins and one to flavonol. Delphinidin 3-glucoside (D3G) was the most frequent anthocyanin interacting with proteins including 11β-HS (-6.59 kcal/mol) forming hydrogen bonds with LYS A:159, GLY A:141, GLY A:94, and ASN A:152. GFAT (-6.38 kcal/mol) interacting through hydrogen bonds with SER A:401, ASP A:427, ASN A: 679, and GLU A:560. PTP (-6.22 kcal/mol) with residues ASP A:48, ARG A:221, LYS A:120, and TYR A:46. RTKs (-6.62 kcal/mol) forming residues with VAL A:1274, and PRO A:1272. followed by cyanidin 3-glucoside (C3G) with proteins 11β-HS (-6.27 kcal/mol) interacting with SER A:12, GLY A94, and TYR A:155, GFAT (-6.10 kcal/mol) with amino acid residues GLN A:977, ASP A: 1056, ASN A:1100, and GLY A:1122. PPARG (-7.32 kcal/mol) with residues VAL A:1274, GLU A:1273, and PRO A1272. Finally, petunidin 3-glucoside (P3G) with proteins 11β-HS (-6.87 kcal/mol) and PTP (-6.24 kcal/mol). Regarding catechin (CAT), it showed an interaction with PPARG (-6.30). The best poses are shown in Figure 5. All proteins showed similar and even better potential than sitagliptin and metformin, two of the most common drugs used as pharmacological therapy in diabetes.

**Table 3:** Free energy of binding interaction of metabolites from blue corn and black bean extracts with target proteins.

Ligand	Free energy of binding (kcal/mol)Target proteins												
	GK	AMPK	11β-HS	IRS	9ILB	DPPIV	CRP	GFAT	PPARG	PTP	RTKs	PKB	IR
C3G	+5.72	-4.18	-6.27	-5.16	+188.92	-4.54	+522.2	-6.10	-7.32	-5.85	-5.95	-5.20	-3.82
C4H	+17.36	-0.20	-5.44	-1.38	+146.78	-2.03	-4.27	-2.87	-1.45	-4.45	-1.20	-3.56	-3.92
C3S	+1.13	+194.43	-3.04	+340.48	+123.71	+0.04	-3.34	-1.12	+14.61	-0.97	-0.34	-1.70	-2.56
DAD	+87.33	+23.84	-6.00	+7.37	+804.47	-1.28	-5.45	-2.56	-0.39	-3.12	-3.74	-3.42	-1.49
P3G	-3.78	-4.52	-4.60	-4.14	+704.48	-1.13	-3.76	-2.38	-1.47	-3.47	-3.33	-2.41	-3.75
AOH	+459.35	+17.13	-5.92	-1.94	+559.53	-2.02	-4.27	-3.51	-0.88	-4.16	-2.01	-2.41	-2.84
CFA	-3.25	-5.08	-4.45	-4.29	-4.53	-3.59	-3.31	-4.64	-5.56	-5.12	-4.45	-4.35	-3.92
CQA	+21.91	+0.36	-5.10	-1.05	+280.23	-1.10	-4.63	-3.08	-1.14	-2.69	-1.04	-2.76	-3.57
CCG	+2.40	+1.63	+1.67	+1.75	+3.74	-0.69	-3.40	+1.43	+88.82	-0.11	-0.67	+0.17	-1.29
CMG	+1.7	+131.03	-4.37	+85.38	+1.29	-0.91	-2.73	-0.64	+24.00	-1.70	-4.72	+0.32	-2.51
PCA	-3.32	-3.53	-3.98	-2.75	-0.30	-2.26	-3.57	-2.94	-2.89	-3.79	-1.66	-3.98	-1.43
FRA	-1.52	-3.64	-4.28	-2.60	+15.92	-2.67	-3.96	-3.11	-3.16	-4.02	-2.88	-3.53	-3.57
Q3G	+427.69	+37.53	-5.18	+46.58	+495.79	-1.85	-4.47	-2.83	+26.43	-4.05	-2.91	-2.62	-2.48
D3G	+8.85	-4.44	-6.59	-5.11	-4.21	-5.33	+528.2	-6.38	-6.88	-6.22	-6.62	-5.34	-3.73
GAA	-2.27	-3.35	-3.76	-2.66	-4.53	-2.43	-3.74	-2.71	-2.65	-3.37	-3.23	-2.80	-4.38
K3G	+168.41	+50.66	-5.34	+14.47	+80.31	-1.92	-4.84	-2.38	+3.10	-4.66	-4.17	-1.81	-4.06
M3G	+12.0	-3.44	-5.48	-5.04	+256.6	-4.82	+494.6	-5.44	-5.66	-5.17	-5.49	-5.01	-2.59
DHA	-2.44	-3.37	-3.36	-2.17	+1.06	-1.72	-3.33	-2.94	-2.90	-3.23	-3.63	-3.32	-0.29
Q3R	-5.11	-4.40	-2.75	-3.66	-2.79	-5.05	-2.22	-3.58	-1.95	-2.75	-2.00	-1.02	-1.03
PTG	+8.90	-4.96	-6.87	-5.47	+277.25	-5.81	+609.64	-5.93	-5.78	-6.24	-6.21	+277.25	-3.75
P3M	+1.13	+160.14	-3.34	+252.03	+977.39	-0.67	-3.17	-1.13	+1.64	-1.29	-1.18	-1.28	-2.73
MYG	+431.71	+46.61	-4.88	+151.67	+1.01	-1.69	-1.90	-2.86	+2.12	-3.94	-1.05	-2.27	-3.80
CAT	-2.39	-5.15	-5.42	-4.15	+69.22	-4.25	+152.3	-5.24	-6.30	-5.20	-4.95	-5.50	-4.12
K3X	-2.02	-1.80	-4.04	-3.63	+184.71	-3.98	-4.75	-3.71	-187	-0–90	--2.61	-3.33	-1.91
SIT	+6.92	-1.88	-5.18	-3.81	+187.74	-4.68	-4.30	-5.77	-4.07	-6.21	-3.60	-4.87	-1.40
MET	-5.32	-3.79	-2.91	-4.56	-3.55	-4.46	-4.38	-3.68	-4.54	-2.28	-2.53	-4.41	-4.11

**Table 4:** Summary of inhibition constant (Ki), and types of molecular interactions of then best interactions, between metabolites from blue corn and black bean with proteins.

Ligand	Protein	Binding energy (kcal/mol)	Ki (μM)	H Bonds	Polar	Hydrophobic	π-π	Cation π
Delphinidin 3-glucoside	11β-HS	-6.59	44.82	-----	O12-ASN152O11-ASN152H7-ASN152O1-TYR155O6-TYR155H4-TYR155O10-TYR155O4-LYS159H2-LYS159	------	C21-TYR155C19-TYR155C8-PHE192C11-PHE192C12-PHE192C15-PHE192	H7-TYR155H4-TYR155H6-TYR155
	GFAT	-6.10	21.23	-----	O9THR375H5-THR375O4-SER401H2-SER401O3-SER401O3-ASP427H1-ASP427O5-ASP427H3-ASP427O9-GLU560	C5-CYS373C4-CYS373C1-CYS373C15-LEU673C20-LEU673C21-LEU673C19-LEU673C15-VAL677	-----	-----
	PTP	-6.22	27.59	O5 - TYR46O5-LYS120O12-ARG221O10-ARG221H3-TYR46H6-CYS215H6-ARG221	O2-TYR46O3-LYS120O7-GLN262O11-GLN266H7-GLN266	C5-TYR46C10-VAL49C11-VAL49C13-VAL49C14-VAL49C17-VAL49C18-VAL49C2-PHE182C1-PHE182C3-PHE182	C9-TYR46C15-PHE182C20-PHE182C12-PHE182	H7-PHE182H4-PHE182H1-PHE182
	RTKs	-6.62	13.96	O6-CYS105604-CYS1056H4-CYS1056H2-CYS1057H4-TYR1122	O11-GLU1280O8-GLU1281O1-GLU1281	-----	-----	H1–HIS1057
Cyanidin 3-glucoside	11β-HS	-6.27	25.17	O3-SER12O4-ASN90O10-TYR155H1-SER11H1-SER12H2-AN90H6-TYR155	O3-SER11O5-SER12H3-SER12O11-TYR155H7-TYR155	C20-MET193	C7-PHE192C8-PHE192C9-PHE192C10-PHE192C11-PHE192C14-PHE192	H7-TYR155
	GFAT	-6.10	33.86	O3-SER12O4-ASN90O10-TYR155H1-SER11H1-SER12H2-ASN90H6-TYR155	O3-SER11O5-SER12H3-SER12O11-TYR155H7-TYR155	C20-MET193	C7-PHE192C8-PHE192C9-PHE192C10-PHE192C11-PHE192C14-PHE192	H7-TYR155
	PPARG	-7.32	4.34	O8-ARG288	O4-ARG288H2-ARG288O6-GLU291H4-GLU291O1-GLU291O10-GLU291H6-GLU291O7-GLU295O10-GLU295H6-GLU295	C19-PRO227C21-PRO227C20-LEU228C16-LEU228C14-ALA292C17-ALA292C18-ALA292C17-MET329C18-MET329C7-LEU333	-----	H5-PHE226
Petunidin 3-O-glucoside	11β-HS	-6.87	9.19	O5-GLY94H3-PHE192	O12-ASN90H7-ASN90H6-ASN90O8-ASN152O7-TYR155	C22-ILE14C9-TYR155C22-VAL188C14-MET193	C10-TYR155C13-TYR155C14-TYR155C17-TYR155C18-TYR155C12-TYR155	-----
	PTP	-6.24	26.55	-----	O3-ARG24H1-ARG24O10-TYR46H5-TYR46O9-ARG47H7-ARG47O12-ASP48H7-ASP48O11-ASP48H6-ASP48H5-SER216O3-GLN262O5-GLN262H3-GLN262	C22-TYR46C7-VAL49C9-VAL49C10-ALA217C13-ALA217C17-ALA217C4-ILE219	C15-TYR46C12-TYR46C14-TYR46C18-TYR46C17-PHE182C18-PHE182	H5-TYR46
Catechin	PPARG	-6.30	24.20	-----	O2-ARG288O1-ARG288O2-ARG288H1-GLU291O3-GLU291H2-GLU291O4-GLU295H3-GLU295	C14-LEU330C2-LEU3333C10-LEU333C14-VAL339C13-ILE341C15-ILE341	-----	H3-PHE226

## 4 Discussion

The aim of our study was to identify the polyphenol content of extracts from black beans and blue corn and elucidate their potential interaction with proteins involved in the pathogenesis of T2D, with the objective to decipher potential molecular mechanisms of action underlying their anti-diabetic effect. In this work we observed that anthocyanins were the major group of polyphenols in both extracts. Regarding blue corn anthocyanins distribution, we did not perform a quantification of each polyphenol but it has been widely published that cyanidin 3-glucoside represents more than 75% of the total anthocyanins in different blue corn samples, while the other cyanidin derivatives are distributed in equal proportions (Aparicio-Fernandez et al., 2005; Bridle and Timberlake, 1997; Mazza and Miniati, 1993). Previous reports with blue corn showed the existence of cyanidin mono-glucosides, malonyl derivatives of peonidin and pelargonidin 3-glycosides as well as p-coumaric and malonic acid moieties (Aparicio-Fernandez et al., 2005; Bridle and Timberlake, 1997; Mazza and Miniati, 1993). In black bean sample, a variety of polyphenols was identified. These results are similar to results found in literature, few flavonols as catechin, quercetin, myricetin and kaempferol were present as aglycones and with glucosides. Phenolic acids as ferulic acid, p-coumaric acid, gallic acid and 3, 5 dihydroxybenzoic acid were found and are aligned with previous reports (de Pascual-Teresa et al., 2002; Del Pozo-Insfran et al., 2006; Díaz-Batalla et al., 2006). Myricetin, kaempferol, and daidzin were found in two black bean varieties. Six anthocyanins were identified in black bean sample, other studies have reported only two anthocyanins in different varieties of black bean (delphinidin 3-glucoside and petunidin 3-glucoside) (Aparicio-Fernandez et al., 2005; Lin et al., 2008).

**Figure 6:**
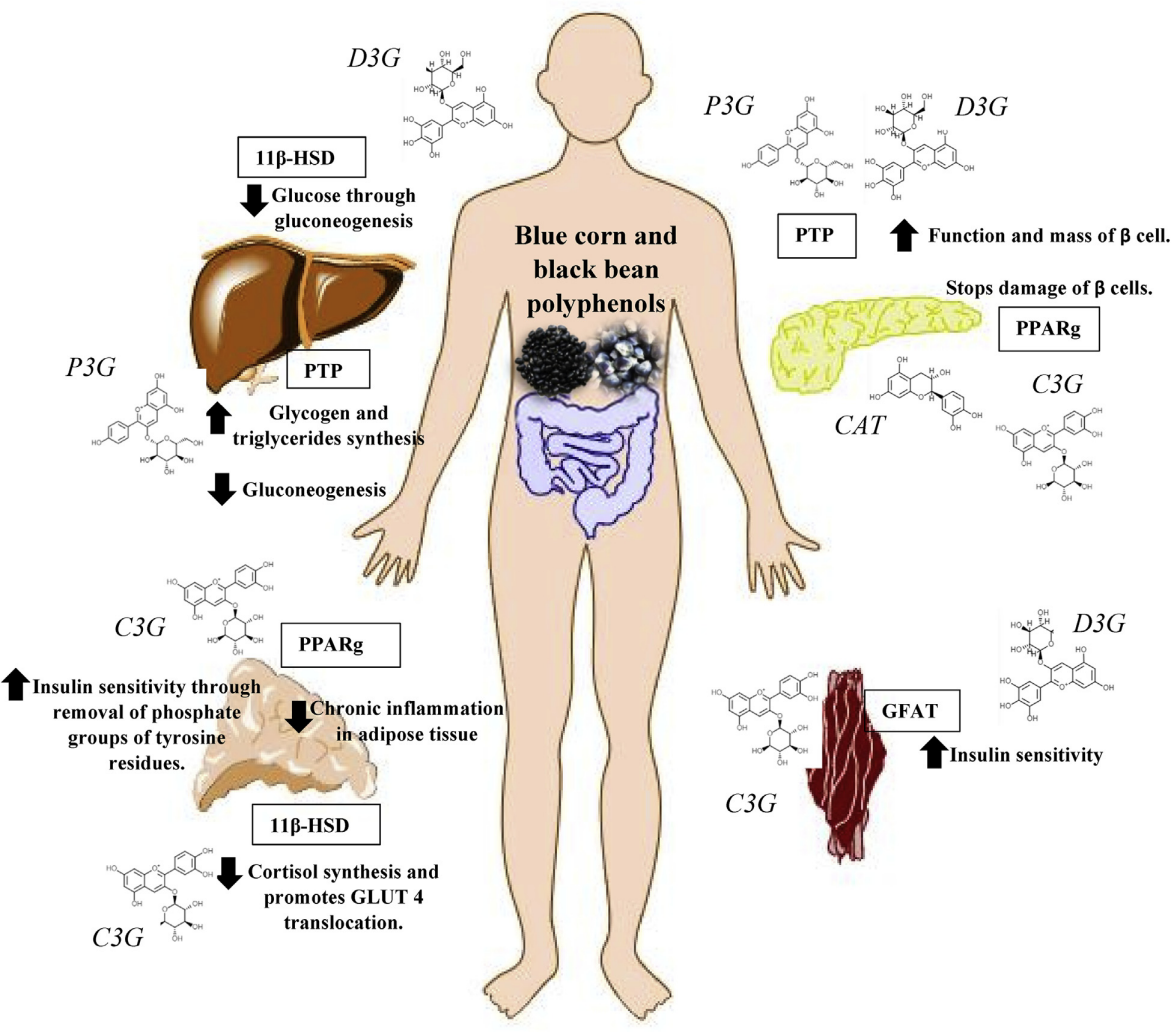
Summary of the potential mechanisms of black bean and blue corn polyphenols on inhibition of target proteins involved in cell signaling of type 2 diabetes mellitus.

A summary of the potential mechanisms of polyphenols is shown in Figure 6, the *in silico* analysis showed that D3G was the metabolite with more interactions, followed by C3G, P3G, and CAT. This result can be explained according to chemical structures of anthocyanins, where D3G has structural characteristics such as double bonds, catechol and oxo groups, two phenolic rings linked by a pyranic ring and major number of hydroxyl groups that produce better bioactivity (Jakobek, 2015; Morales-Flores et al., 2015). Molecular docking suggests that D3G and P3G could inhibit protein tyrosine phosphatase (PTP) that plays important roles in tyrosine phosphorylation and dephosphorylation, a basic mechanism of cell growth and differentiation (Patel et al., 2014). Insulin is a key regulator of liver homeostasis, dephosphorylation of the insulin receptor (IR) by PTP's during its internalization, produces gluconeogenesis inhibition and promotes the synthesis of glycogen and triglycerides. Loss of this tight regulation produces to increased hepatic insulin signaling, improved insulin suppression of glucose production, decreased serum and hepatic triglyceride and cholesterol levels, and less protection against reactive oxygen species in protein phosphorylation results in over or under activation of main signaling pathways (Gurzov et al., 2015). In pancreas, if PTP's expression is increased, insulin receptor substrate lack improves β-cell function and stimulates compensatory islet growth leading to a dysfunction of insulin secretion (Sun et al., 2016). In muscle and adipose tissue, the expression of particular PTPs is related with the development of T2D. In muscle cytosol from obese nondiabetic patients, PTP was increased compared to lean individuals (Gurzov et al., 2015). Our results suggest that both anthocyanins could improve glucose levels, insulin metabolism, and fat accumulation by binding to this protein. There is not enough evidence that supports mechanisms of actions of D3G and P3G on PTP's inhibition, but few studies reported that flavonoids isolated from different plants possess PTP inhibitory activity. Molecular docking of PTP protein with epigallocatechin and caffeic acid extracted from *Geranium collinum* showed good binding energy. However, mechanisms were not evidenced (Tamrakar et al., 2014). An *in vivo* study with Cudrania tricuspidata (CTe) leaves rich in polyphenols showed a strong inhibitory effect on PTP activity. The extract decreased levels of aspartate aminotransferase and alanine aminotransferase, triglycerides, body weight, total cholesterol, fat, blood glucose and, improved insulin secretion. CTe extracts showed an effect in liver tissue increasing the phosphorylation of IRS-1 and Akt proteins (Kim et al., 2016b, Kim et al., 2016a). Therefore, these studies suggest that polyphenols linked to PTP, decrease the activity of the latter, and prevent the development of T2DM and its complications.

C3G and D3G showed potential binding capacity with 11β-HS, hormone that catalyzes the interconversion of the glucocorticoids, cortisone, and cortisol in humans. These hormones are key regulators of glucose and lipid homeostasis maintenance, high levels of glucocorticoids can produce insulin resistance by reducing insulin-dependent glucose uptake, improving hepatic gluconeogenesis and inhibiting insulin secretion from pancreatic cells. They not only stimulate glucose production in the liver, these hormones increase lipolysis and fatty acid mobilization, as well as antagonize insulin-mediated glucose uptake in peripheral tissues such as adipose and muscle (Geer et al., 2014). Individuals with systemic glucocorticoid excess develop visceral obesity, insulin resistance, glucose intolerance, dyslipidemia, hypertension, etc (Hollis and Huber, 2011). Even though there is not much research on polyphenols effect on 11b-HSD1 protein, a study with Italian espresso coffee extract showed the inhibition of recombinant and endogenous 11b-HSD1 dependent cortisol development activity, the prevention of the consequent nuclear translocation of the glucocorticoid receptor and the eradication of glucocorticoid-induced expression of the enzyme phosphoenolpyruvate carboxykinase (Atanasov et al., 2006). Another study demonstrated that catechins and (-) Epigallocatechin gallate from green tea, exhibited the highest inhibitory potential of 11b-HSD1 activity with an IC50 value of 3.749 mg of dried tea leaves per ml. The authors associated the inhibition of 11b-HSD1 with the decrease of hepatic gluconeogenesis (Hintzpeter et al., 2014).

We also observed that CAT and C3G present potential interaction with PPARγ. PPARγ is a main regulator of cellular differentiation, lipid and glucose homeostasis. In adipose tissue PPARγ is most abundantly expressed were it regulates the transcription of the adiponectin gene. In the pancreatic beta-cell it is also expressed and is associated with an abnormal increase of beta-cell mass (Buse et al., 2011). Some of the polyphenols reported with anti-inflammatory effects are capsaicin, resveratrol, genistein, and daidzein, previous research suggest that these polyphenols are involved in direct activation of PPARg (Aryaeian et al., 2017). Other phytochemicals such as c-baptigenin, hesperidin, 20 hydroxy chalcone and quercetin have also been demonstrated to interact with PPARg ligands (Scazzocchio et al., 2011; Tian et al., 2013). The interest in the study of PPARg as gene with therapeutic utility, is due to their potential as a metabolic regulator of peripheral organs and tissues, such as adipose tissue. Upregulation of PPARg expression/activity has been reported to improve insulin sensitivity and glucose uptake through GLUT 4 translocation pathway in human adipocytes and in animal models of diabetes. The increase in glucose uptake was associated with enhanced GLUT 4 translocation and adiponectin secretion, which was caused by the increased activity of PPARg induced by polyphenols (Buse et al., 2011). D3G and C3G showed the inhibition of GFAT, this enzyme is one of the most important of the HBP pathway and catalyzes the amidation of fructose-6-phosphate to glucosamine6-phosphate in the presence of glutamine. Glucose fluidity through this pathway is considered as a system of nutrient sensing and HBP is one of pathways through which hyperglycemia mediates peripheral insulin resistance and diabetic complications. However, it is still unclear how the signal deriving from increased HBP flux produces insulin resistance. The increased activity of GFAT has been involved in insulin resistance in different experimental models (Srinivasan et al., 2007; Zhang et al., 2004), but less is known about polyphenol effects in GFAT. Srinivasan (2014), investigated glucose metabolism, lipid profile, blood pressure, and expression of genes related to glucose and lipid metabolism. Likewise, they studied the beneficial effects of a diet enriched with anthocyanins bilberries or blackcurrants on these pathways. Thus, they showed that constant bilberry consumption decreased total and LDL-cholesterol levels. However, they did not find changes in HDL-cholesterol levels in ZDF rats. Nevertheless, neither of the fruits ameliorated the development of T2DM, and differences in expression levels of genes related to glucose metabolism. On the other hand, they observed differential gene expression in hepatic tissue, which can be explained by the abundant distribution of anthocyanins in liver (Srinivasan, 2014).

## 5 Conclusion

Our study demonstrated from an *in silico* approach that polyphenols found in blue corn and black bean extracts, especially anthocyanins, have the potential to interact and modulate the activity of proteins involved in the main pathways of type 2 diabetes mellitus such as insulin secretion, insulin resistance and carbohydrate absorption. This suggests that both foods rich in polyphenols could represent a good source of bioactive compounds that can be used as an alternative for the prevention of metabolic disorders and associated diseases. Nevertheless, further investigations with different approaches in animal models and humans should be carried out to confirm these findings and gain a better understanding of the mechanisms of these polyphenols underlying their metabolic health properties.

## Declarations

### Author contribution statement

Karla Damián-Medina: Conceived and designed the experiments; Performed the experiments; Analyzed and interpreted the data; Wrote the paper.

Yolanda Salinas-Moreno, Alba Vallejo-Cardona: Performed the experiments; Contributed reagents, materials, analysis tools or data.

Dragan Milenkovic, Luis Figueroa-Yañez: Analyzed and interpreted the data; Contributed reagents, materials, analysis tools or data.

Erika Marino-Marmolejo, Inocencio Higuera-Ciapara: Analyzed and interpreted the data.

Eugenia Lugo-Cervantes: Conceived and designed the experiments; Analyzed and interpreted the data; Contributed reagents, materials, analysis tools or data.

### Funding statement

This work was supported by the project FORDECYT-CONACYT (Mexican National Council of Science and Technology) (No. 2017-292474).

### Competing interest statement

The authors declare no conflict of interest.

### Additional information

No additional information is available for this paper.
